# Supplementary material for: Mechanisms Explaining Transitions between Tonic and Phasic Firing in Neuronal Populations as Predicted by a Low Dimensional Firing Rate Model
Source: PLoS One. 2010 Sep 22;5(9):e12695. doi: 10.1371/journal.pone.0012695 (PMC2943909; doi:10.1371/journal.pone.0012695)
Supplement: Appendix S1 — (0.18 MB PDF) [file pone.0012695.s001.pdf]

Supplementary Material – Text S1

To produce the **membrane potential illustration of single cell bursting in Figure 1A**, we have used the basic biophysical model for bursting neurons proposed by AvRon et al. [29], based on adaptation of the K channel conductance:

$$
\begin{aligned}
C_m \frac{dV}{dt} &= I - I_{Na} - I_K - I_L \qquad (8)\\
\frac{dW}{dt} &= \frac{W_\infty(V) - W}{\tau_w(V)}\\
\frac{d\bar{g}_K}{dt} &= S(V)(V - V_{rest}) - d(\bar{g}_K - \bar{g}_K^{(rest)})
\end{aligned}
$$

where

$$
\begin{aligned}
I_{Na} &= \bar{g}_{Na} m_\infty^3(V)(1-W)(V - V_{Na})\\
I_K &= \bar{g}_K (W/s)^4 (V - V_K)\\
I_L &= \bar{g}_L (V - V_L)
\end{aligned}
$$

and

$$
\begin{aligned}
W_\infty(V) &= \left(1 + \exp[-2a^{(w)}(V - V_{1/2}^{(w)})]\right)^{-1}\\
m_\infty(V) &= \left(1 + \exp[-2a^{(m)}(V - V_{1/2}^{(m)}]\right)^{-1}\\
\tau_w(V) &= \bar{\lambda}\left(\exp[a^{(w)}(V - V_{1/2}^{(w)})] + \exp[-a^{(w)}(V - V_{1/2}^{(w)})]\right)\\
S(V) &= S \cdot \left(1 + \exp[-2a^{(s)}(V - V_{1/2}^{(s)})]\right)
\end{aligned}
$$

We used the following parameter values: $V_{Na} = 55$ mV, $V_K = -72$ mV, $V_L = -49.3$ mV, $V_{rest} = -56$ mV, $\bar{g}_{Na} = 120$ mS/cm^2, $\bar{g}_L = 0.3$ mS/cm^2, $\bar{g}_K^{(rest)} = 8$ mS/cm^2, $C_m = 1$ μF/cm^2, $s = 1$, $a^{(w)} = 0.045$, $V_{1/2}^{(w)} = -55$ mV, $a^{(m)} = 0.05$, $V_{1/2}^{(m)} = -33$ mV, $a^{(s)} = 0.2$, $V_{1/2}^{(s)} = -20$ mV, $\lambda = 0.5$, $S = 0.04$, $p = 0.008$.

To produce the **mean field firing rate illustration of a bursting neural population in Figure 1B**, we used the two-dimensional model described in this paper:

$$
\begin{aligned}
\tau_F \frac{dF}{dt} &= -F + [F_{\max} - F]\mathcal{S}[aF - b_{\max} b + P] \qquad (9)\\
\tau_b \frac{db}{dt} &= b_\infty(F) - b
\end{aligned}
$$

where

$$
\begin{aligned}
\mathcal{S}(Y) &= \frac{1}{1 + \exp[-k_S(y - y_S)]}\\
b_\infty(F) &= \frac{1}{1 + \exp\left[-k_b(F - F_b)\right]}
\end{aligned}
$$

We used the following parameter values: $F_{\max} = 400$ Hz, $a = 0.685$, $P = 100$, $b_{\max} = 160$, $\tau_F = 2.5$ msec, $\tau_b = 66$ msec, $k_S = 0.2$, $y_S = 80$ Hz, $k_b = 0.025$, $F_b = 60$ Hz.

To justify our mean field approach to bursting in this paper, we would like to discuss here a few straight forward advantages of this model versus using a network of coupled minimal bursting models. In our future work, we will investigate further the possibility of using coupled FitzHugh-Nagumo systems to model interactions between populations, rather than the dynamics of coupling of individual cells.

The first aspect we discuss is the computational advantage residing in the mean approach over the network of individual cells. Modeling a network of n such cells would require coupling n minimal bursting systems (8), hence a number $3n$ of coupled, highly nonlinear, ordinary differential equations. Even assuming simple electrical and identical mutual coupling and a small network (e.g., a realistic network may vary between 2-50 cells [49]), the corresponding $3n$-dimensional ODE will still have rather large proportions:

$$
\begin{aligned}
C_m \frac{dV(j)}{dt} &= I + \sum_{k=1}^{n} g[V(k) - V(j)] - I_{Na} - I_K - I_L \qquad (10)\\
\frac{dW(j)}{dt} &= \frac{W_\infty(V) - W}{\tau_w(V)}\\
\frac{d\bar{g}_K(j)}{dt} &= S(V)(V - V_{rest}) - d(\bar{g}_K - \bar{g}_K^{(rest)})
\end{aligned}
$$

Here g is the gap junctional conductance between each two cells [33]. One may count the operations necessary to find numerically a solution for such a system employing an Euler approximation algorithm (which we use as a simple example, comparable with methods such as Runge-Kutta, used by Matlab's ode45, and by other ODE solvers). For the time interval T and N integration steps of length $h = T/N$, the population approach will resolve the computation with a number of operations $O_1 = 12A + 7M + 2E$; meanwhile, system (8) will need $O_2 = 24A + 29M + 5E$ operations, implying that the coupled-cells system will need $\gtrsim nO_2$ operations (where we denoted by M the number of multiplications, by A the number of additions and by E the number of exponentials). In real time terms, this represents the difference between a few seconds and a few minutes (the latter increasing, of course, with the number of coupled cells). If either model is meant to be used as a building unit in constructing larger-field networks (connecting mid-brain structures with each other and with external modulatory structures), this difference will increase in proportion to the number of units, and thus the computational time aspect could become very important.

Secondly, we look at some single cell phenomena ignored by the mean filed approach, and we discuss whether this simplification may negatively affect the results and their interpretations, or if it rather provides a convenient schematic representation of the physiology. Indeed, the processes of modeling (theoretically) or recording (experimentally) the overall behavior of an entire neural population overlook certain dynamic rhythms at the single-cell level. Let's consider, for example, a small network of $n = 5$ cells, with identical properties – i.e., identical parameters in all equations of the coupled system (10) – but entering the bursting mode with a variety of initial states (initial conditions for the variables (V, W, g_K)). As parameter values, we considered the same values as the ones given for the system (8) at the start of this section; in addition, we set the coupling conductance $g = 0.0005$ mS/cm^2.

As shown in Figure 9A, we observe an immediate segregation of the behavior of the $n = 5$ cells into two synchronized rhythms shifted in phase, one rhythm followed by $n_1 = 3$ cells, and the other by the remaining $n_2 = 2$ cells. So the dynamics of the population is not homogeneous in the context of the timing of individual action potentials. However, considering a bigger picture, it becomes clear that these rhythmic spike are, in both subsets of cells, part of a well-defined intra-burst interval, which in turn will repeat periodically, separated by inter-burst quiescence (Figure 9B). Moreover, although the two population subsets are not synchronized at the level of their spike-times, the starting and ceasing of bursting appears to be identical for the two, suggesting a synchronization in rhythmic bursting that

justifies our firing-rate based "homogeneous" representation of the population. While, in this light, the approach is naturally inappropriate for representing single cell behavior, for the purpose of studies which model results from *in vivo* electrode recordings, the rhythmic bursting activity is what one would hear in a recording, and whose dynamics one tries to model and understand. We suggest that the model can therefore support such inhomogeneities in the population activity; this is important, since synchronization is observed experimentally only to a certain degree, and assuming it in a theoretical model would be unrealistic.

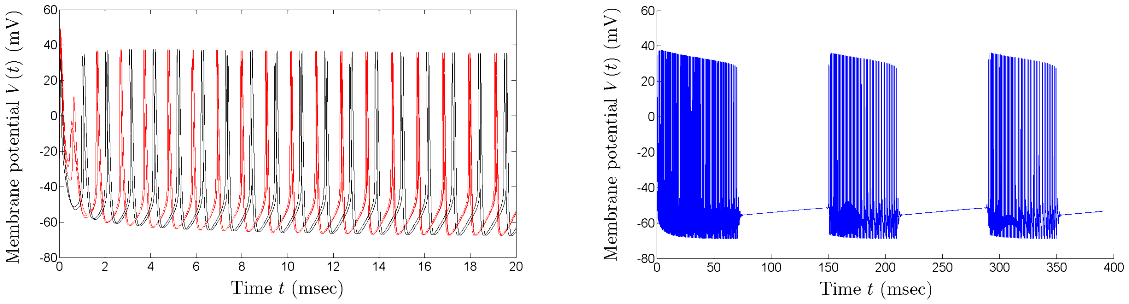

Figure 9. Behavior of a small network of electrically coupled single cell bursters. A. The plots represent the time evolution of the membrane potential $V(t)$ of each of $n = 5$ cells coupled electrically to form a small network. The cells are identical, and exhibit individual bursting properties, as described by system (8), with the parameters already listed and gap junctional coupling g. Based only on the initial conditions of the components, the network segregates into two out of phase, synchronized subsets (one composed of 3 cells, whose plots are shown in red, and the other composed of 2 cells, whose plots are shown in black). **B.** The representation of the same time evolutions for a substantially longer time reveals repetitive bursting, and sows that, despite the segregation into two individual spike-time rhythms, the bursting and inter-bursting intervals coincide for all five cells. This suggests a mean field approach to be appropriate in the context of studying the population bursting dynamics.
