# Supplementary material for: Mechanisms Explaining Transitions between Tonic and Phasic Firing in Neuronal Populations as Predicted by a Low Dimensional Firing Rate Model
Source: PLoS One. 2010 Sep 22;5(9):e12695. doi: 10.1371/journal.pone.0012695 (PMC2943909; doi:10.1371/journal.pone.0012695)
Supplement: Appendix S2 — (0.22 MB PDF) [file pone.0012695.s002.pdf]

Supplementary Material – Text S2

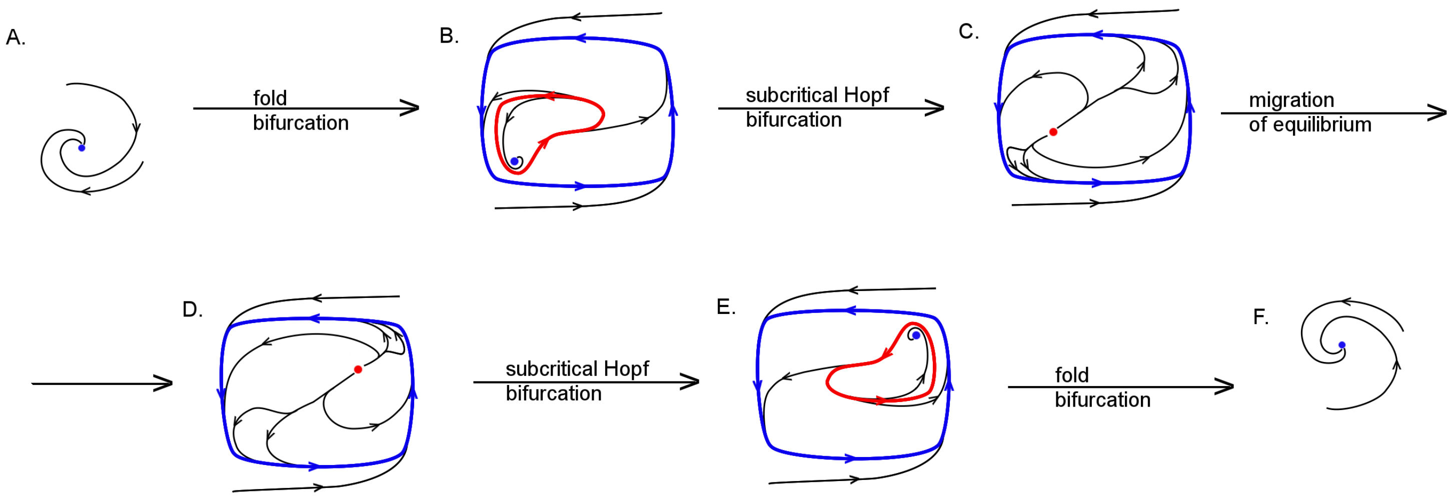

Figure 10. A succession of four qualitative transitions (bifurcations) undergone by the system (6)-(7) **as one parameter is varied.** In this case, F_b was increased from 0 to 200 Hz, while the other system parameters were held fixed (in particular, the intrinsic amplification was fixed to $a = 0.5$; compare with Figure 3A in the main text). We illustrate the main features of the phase-plane for each interval between two consecutive bifurcations. For small values of F_b, there is a globally attracting equilibrium, situated in the low firing range (**A**). Increasing F_b past the first critical value, makes the system undergo a fold bifurcation: one half-stable cycle appears around the equilibrium. Under further increases in F_b, this cycle separates into a large attracting cycle (blue cycle in **B**), and a small repelling cycle (red cycle in **B**). The unstable cycle is the boundary between the basins of attraction of the stable equilibrium and of the stable cycle. Continuing to increase F_b maintains for a short while this bistability regime, with no more qualitative changes; however, quantitatively, while the large stable cycle only changes shape slightly under increments of F_b, the small unstable cycle gradually shrinks around the stable equilibrium. At the next critical value for F_b, the unstable cycle becomes so small that is swallows up the equilibrium and disappears (subcritical Hopf bifurcation), changing the stability of the equilibrium in the process. The system has now an unstable equilibrium surrounded by a large stable cycle (**C**). Under further increments of F_b, the unstable equilibrium will travel inside the cycle from its lower left, corresponding to low firing rates, towards the upper right, corresponding to high firing rates (**D**). Reaching the third critical value of F_b produces another (reverse) subcritical Hopf bifurcation, in which the small repelling cycle reappears around the equilibrium (although now in a different position), while changing again the stability of the equilibrium (**E**). Increasing F_b past this point allows the unstable cycle to enlarge, and eventually collide with the surrounding stable cycle, such that they both disappear – through a fourth bifurcation (a second fold bifurcation). The only invariant feature remaining is the stable equilibrium, now situated in the high firing range (**F**).
