# Supplementary material for: Mechanisms Explaining Transitions between Tonic and Phasic Firing in Neuronal Populations as Predicted by a Low Dimensional Firing Rate Model
Source: PLoS One. 2010 Sep 22;5(9):e12695. doi: 10.1371/journal.pone.0012695 (PMC2943909; doi:10.1371/journal.pone.0012695)
Supplement: Appendix S3 — (0.27 MB PDF) [file pone.0012695.s003.pdf]

Supplementary Material – Text S3

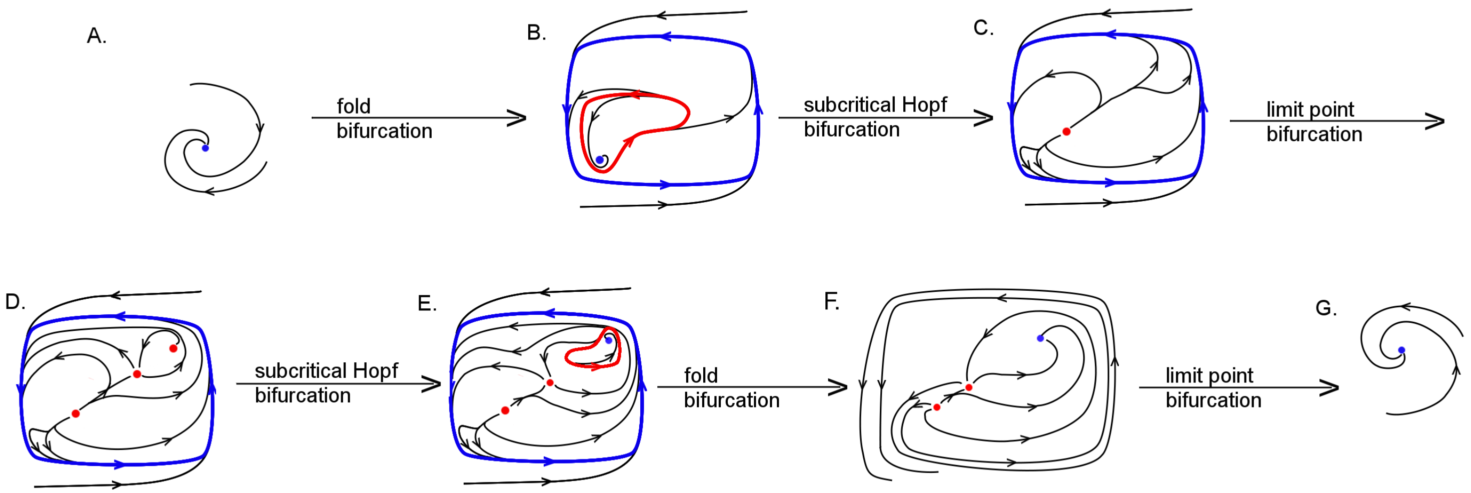

Figure 11. A succession of six qualitative transitions undergone by the system (6)-(7) **as one parameter is varied.** Here, F_b is increased from 0 to 200 Hz, while the other parameters are held fixed (in particular, $a = 0.75$; compare with Figure 3B in the main text). As before, we illustrate the main features of the phase-plane for each interval between two consecutive bifurcations. For small values of F_b, analogously with Figure 1, there is a globally attracting equilibrium, situated in the low firing range (**A**). Increasing F_b through the next two critical values, makes the system undergo a similar fold bifurcation (**B**), then a similar subcritical Hopf bifurcation (**C**) as in Figure 1B . From this point on, however, increasing F_b produces a succession of bifurcations significantly different from the case of $a = 0.5$. At the third critical value for F_b, two additional (both unstable) equilibria appear inside the large stable cycle: an unstable spiral and a saddle. This critical point is a limit-point (or, by a different terminology, a saddle node) bifurcation (**D**). The fourth critical value of F_b brings a second subcritical Hopf bifurcation, in which a small repelling cycle appears around the unstable spiral, changing it into a locally stable spiral (**E**) and bounding its attraction basin. Increasing F_b past this point allows the unstable cycle to enlarge, and eventually collide into the surrounding stable cycle (before it could crash into the nearby saddle point), such that both cycles disappear; this constitutes the fifth bifurcation – fold bifurcation, slightly more complex than the analogous one in Figure 3A (**F**). The three equilibria (a stable spiral, a saddle and an unstable spiral) all survive this transition. Increasing F_b further, however, causes the two unstable equilibria to collide and disappear (the sixth, limit point, bifurcation). Past this last critical value of F_b, only the high firing stable spiral remains (**G**).
