# Supplementary material for: Mechanisms Explaining Transitions between Tonic and Phasic Firing in Neuronal Populations as Predicted by a Low Dimensional Firing Rate Model
Source: PLoS One. 2010 Sep 22;5(9):e12695. doi: 10.1371/journal.pone.0012695 (PMC2943909; doi:10.1371/journal.pone.0012695)
Supplement: Appendix S4 — (0.15 MB PDF) [file pone.0012695.s004.pdf]

Supplementary Material – Text S4

Examples of the bifurcations displayed by the system as two parameters are varied simultaneously are shown in Figures 12 and 13. Figure 12 illustrates the bifurcation curves of the system as the extrinsic input rate P and its weight a are varied. Figure 4B shows the Hopf limit point transitions that occur on each steady state curve (i.e., for each distinct value of a), as the parameter P is increased. This means that, if a is varied, the Hopf points will describe a *Hopf curve*, and the limit points will describe a *limit point curve*. In Figure 12A, these are shown in red (the Hopf curve) and green (the limit point curve); compare with Figure 4B, where a few of these Hopf points are plotted as red stars, and a few of the limit points are plotted as green stars. The bifurcation curves are drawn over the whole parameter domain, to provide a complete illustration of the underlying mathematics, but the portions corresponding to unbiological values of a (i.e., $a \notin [0, 1]$) are dotted. A clearer interpretation can be formulated if one observes the same curves plotted in the two-dimensional (P, a) parameter plane, in panel B of the same figure. The red Hopf curve encloses a compact region $\mathcal{K}$ in this parameter plane, which corresponds to the bursting regime. Triggering bursting corresponds to a parameter path which enters $\mathcal{K}$, while cessation of bursting is delivered by the parameter path leaving $\mathcal{K}$. Similar features are found in the (P, F_b) and (a, F_b) parameter planes, as shown in Figure 13, A and B.

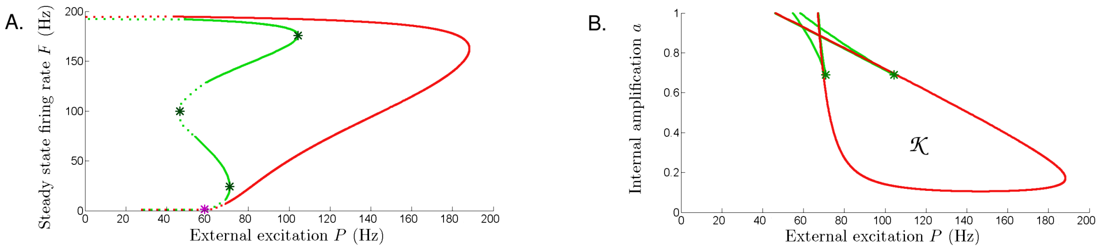

Figure 12. Simultaneous variation of two parameters. A. Bifurcation diagram showing the evolutions of the positions of limit points and of the Hopf points as both parameters a and P change. The limit curve is drawn in green, and the Hopf curve is in red. The curves are dotted outside of the $[0, 1]$ interval for a. The stars mark codimension 2 bifurcations: Bogdanov-Takens (purple star), and cusp (dark green stars). See also Figure 4B. **B.** The same two curves are illustrated in the parameter plane (P, a). Fixed parameters: k_S=0.2, k_b=0.025, y_S=80, $b_{\max}$=160, τ_F=2.5 msec, τ_b=33 msec.

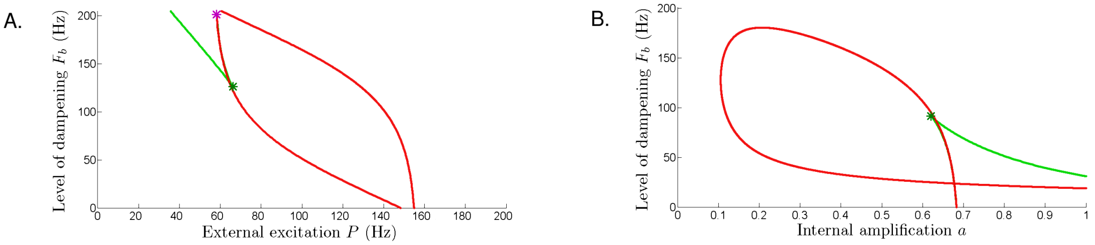

Figure 13. Bifurcation diagrams illustrating the bursting regions in two-dimensional parameter planes. A. Parameter plane (P, F_b). **B.** Parameter plane (a, F_b). Fixed parameters: k_S=0.2 sec, k_b=0.025 sec, y_S=80, $b_{\max}$=160, τ_F=2.5 msec, τ_b=33 msec.
